# First satellite tracks of neonate sea turtles redefine the ‘lost years’ oceanic niche

**DOI:** 10.1098/rspb.2013.3039

**Published:** 2014-04-22

**Authors:** Katherine L. Mansfield, Jeanette Wyneken, Warren P. Porter, Jiangang Luo

**Affiliations:** 1 Department of Biology, University of Central Florida, Orlando, FL 32816, USA; 2 Southeast Fisheries Science Center, National Marine Fisheries Service, 75 Virginia Beach Drive, Miami, FL 33149, USA; 3 Department of Biological Sciences, Florida Atlantic University, Boca Raton, FL 33431, USA; 4 Department of Zoology, University of Wisconsin Madison, Madison, WI 53706, USA; 5 Division of Marine Biology and Fisheries, Rosenstiel School of Marine and Atmospheric Science, University of Miami, Miami, FL 33149, USA

**Keywords:** sea turtle ‘lost years’, ocean migration, satellite telemetry, oceanic stage sea turtles, *Caretta caretta*, thermal niche

## Abstract

Few at-sea behavioural data exist for oceanic-stage neonate sea turtles, a life-stage commonly referred to as the sea turtle ‘lost years’. Historically, the long-term tracking of small, fast-growing organisms in the open ocean was logistically or technologically impossible. Here, we provide the first long-term satellite tracks of neonate sea turtles. Loggerheads (*Caretta caretta*) were remotely tracked in the Atlantic Ocean using small solar-powered satellite transmitters. We show that oceanic-stage turtles (i) rarely travel in Continental Shelf waters, (ii) frequently depart the currents associated with the North Atlantic Subtropical Gyre, (iii) travel quickly when in Gyre currents, and (iv) select sea surface habitats that are likely to provide a thermal benefit or refuge to young sea turtles, supporting growth, foraging and survival. Our satellite tracks help define Atlantic loggerhead nursery grounds and early loggerhead habitat use, allowing us to re-examine sea turtle ‘lost years’ paradigms.

## 1. Introduction

Classic sea turtle life-history models assume discrete shifts in habitat use during different life stages [1–3]. Sea turtles hatch from nests on coastal beaches, enter near-shore waters and swim offshore, transitioning to oceanic habitats where they remain for a minimum of 1–2 years [1–6]. Known as the sea turtle ‘lost years’, few data exist on the in-water behaviour of young, oceanic-stage sea turtles [7]. These knowledge gaps reflect the logistical and technological limitations of observing small, fast-growing, migratory species in the open ocean. Rare sightings, at-sea collections [1,4,8], genetic sampling [9] and spatially discrete size distributions of loggerhead turtles [10] resulted in long-standing hypotheses regarding oceanic-stage sea turtle dispersal and behaviour. These include the hypotheses that neonate Atlantic loggerhead turtles:

(1) transition to and remain offshore in oceanic waters, away from predator-rich Continental Shelf waters [2–4];
(2) are passive drifters that entrain within currents associated with the North Atlantic Subtropical Gyre [1,4]; and
(3) occupy sea surface habitats [1,4,8,11] and associate with floating *Sargassum* communities [1,11,12].

Carr [1] hypothesized that loggerhead hatchlings from eastern Florida (USA) nesting beaches swim offshore and enter the North Atlantic Subtropical Gyre (NASG) via the southern Gulf Stream. Theoretically, turtles are passively transported across the North Atlantic to eastern Atlantic waters and are known to associate with floating *Sargassum* communities for predator refuge and food availability [1,4,11,12]. Size distributions of oceanic loggerheads from the eastern Atlantic (Azores, Cape Verde, Madeira), when compared with those along the Atlantic seaboard, support the hypothesis of a long-term, unidirectional, gyre-based, developmental migration [1,4,5,9,10,13]. Loggerheads found in the eastern Atlantic are genetically linked to nesting assemblages along the western Atlantic coast [9,14]. Laboratory studies demonstrate that hatchlings orient appropriately to remain within the NASG when exposed to magnetic fields replicating those fields found at discrete regions along this system [15]. Travel time within the NASG is generally assumed to be correlated to surface current speeds—similar to drift bottles; Carr [1,4] noted that passively drifting loggerheads within the NASG would require 235 days to traverse from Florida natal beaches to the Azores. However, no actual long-term turtle movements or travel times had been directly observed.

Temperature influences poikilothermic turtle growth, movement, feeding behaviour, physiology and immune competence [16,17]. Experimental tests of hatchling loggerheads during their post-hatching frenzy found that swimming activity decreased in 30°C water and locomotor coordination was lost in waters above 33°C [18]. At cool temperatures (less than 10°C), smaller sea turtles are vulnerable to hypothermic stunning [19,20]. Despite its importance, the thermal environment encountered by oceanic-stage loggerheads has not been directly measured. The significance of *Sargassum* communities as refuge, foraging and early developmental habitat for oceanic juvenile loggerheads is well documented [4,14]. Yet an important function of this surface-based habitat has been overlooked: the thermal benefit of associating with these communities.

Here, we provide the first long-term, at-sea movement data for small, oceanic-stage loggerhead sea turtles. Using novel satellite telemetry methods [7], we remotely tracked neonate loggerheads in the Atlantic Ocean. By characterizing the turtles’ offshore dispersal, thermal niche and habitat characteristics, we re-examine the long-standing hatchling dispersal paradigms and hypotheses associated with the sea turtle ‘lost years’. Specifically, we test whether oceanic-stage loggerhead sea turtles (i) remain exclusively offshore within oceanic (non-neritic) waters, (ii) entrain within the currents associated with the North Atlantic Subtropical Gyre as part of a unidirectional developmental migration and (iii) occupy sea surface habitats. Finally, (iv) we test whether occupying sea surface habitats or association with *Sargassum* communities would confer thermal benefits to small, oceanic-stage sea turtles.

## 2. Material and methods

### (a) Turtle data and movement analyses

Microwave Telemetry's PTT-100 9.5 g solar-powered satellite transmitters were used to track the at-sea movements of 17 neonate loggerhead sea turtles collected from nests along the southeast coast of Florida, and laboratory-reared to release size (300–720 g, 11–18 cm straight carapace length) and age (3.5–9 months old). Tag coloration matched that of a typical loggerhead carapace (brown). Tags were adhered to turtles’ carapaces using a flexible acrylic–silicone–neoprene attachment described by Mansfield *et al*. [7]. Tag duty cycle was programmed to 10 h on, 48 h off per manufacturer requirements—tags required 48 h of solar charging. All turtles were released in the Gulf Stream within floating *Sargassum* mats approximately 18.5 km offshore of their natal beaches (near 26.9° N latitude, 79.5° W longitude). Using the Argos satellite data processing system and Kalman filtering algorithm, transmitter data were filtered based on accuracy of transmission using Argos location codes (LC) 3–0, A and B [21]. Location data were tested for spatial randomness and orientation using circular point and Raleigh's Z statistics (ArcView v. 3.2, AMAE ext.; *α* < 0.05). Mean orientation was determined for locations occurring below and above 35° N latitude, roughly corresponding to the replicated magnetic field locations tested on captive hatchlings by Lohmann & Lohmann [22].

### (b) Thermal ecology and habitat use

Mean daily ambient temperature (°C; ±0.33°C accuracy per manufacturer specifications) and solar cell charge (volt; ±0.02 V accuracy) were collected from transmitter sensor data. Bathymetry data and MODIS 9 km resolution daily sea surface temperatures (SSTs) were extracted using the Satellite Tracking and Analysis Tool (STAT) [23]. Additional SST and bathymetry data were derived using the Global Hybrid Coordinate Ocean Model (HYCOM + NCODA Global 1/12° Analysis; 7 km resolution) and 2 min Gridded Global Relief Data (ETOPO2 v. 2).

To characterize time spent in association with oceanographic features such as the Gulf Stream and other currents or meso-scale eddies, the Kalman-filtered tracks were regularized to a frequency of 6 h intervals, using piecewise Bézier interpolation methods similar to Tremblay *et al*. [24], but modified with the algorithm by Lars Jensen (http://ljensen.com/bezier/). To determine turtle association with eddy features, we compared the 6 h interpolated turtle tracks with daily current vector maps from HYCOM model output for all individuals and tabulated the number of track days associated with each of the three features: (i) main Gulf Stream (main part of Gulf Stream not including eddy features), (ii) eddy feature (defined as a current vector group forming a circular pattern) and (iii) other areas not included in (i) and (ii).

To determine whether turtles remained at the sea surface, and to characterize the thermal environment encountered by the turtles, we used four complementary approaches. (i) We tested for differences among ambient (transmitter-derived) versus satellite- (MODIS) and model-derived (HYCOM) temperatures encountered by the turtles using the Mann–Whitney *U*-test (*α* < 0.05). (ii) We characterized the Argos location accuracy and tags’ solar charge rates in order to determine relative exposure of the tags to air and direct sunlight. The satellite tags do not transmit unless exposed to air. Longer periods of air exposure allow longer periods of communication between tags and overhead satellites, thereby increasing the accuracy of the transmitted location data [7,25,26]. Mansfield *et al*. [7] showed that the power output from comparable solar cells declined with depths as shallow as 30 cm; power output was one-seventh that of solar cells left to charge at 5 cm depths. Thus, we infer that higher Argos location accuracies and greater charge rates relate to longer periods of transmitter exposure to air and to sunlight, enabling the tags to effectively communicate with overhead satellites or exposing tags to the solar energy required to successfully recharge. We use these data as a proxy to determine whether the turtles occupied sea surface habitats. (iii) To determine whether exposure to direct solar energy could influence temperatures encountered by neonate sea turtles (e.g. ambient temperatures recorded by the satellite tags), we measured the solar reflectivity of the Microwave Telemetry 9.5 g PTTs solar-powered satellite tags, loggerhead carapaces and mats of fresh *Sargassum* spp*.* collected from waters offshore of southeast Florida. Solar reflectivity measurements represent the fraction of incident radiation reflected rather than absorbed by a surface or substrate. Measurements were collected in a dark room using a portable ASD spectroradiometer set to a spectral range of 350–2500 nm (10 nm resolution; http://www.asdi.com/products/fieldspec-spectroradiometers). Measurements were made with a 1 cm diameter sensor window that delivered a white light source to illuminate the test object. A white reference standard was used to determine 100% reflectivity across the full spectrum; the sensor head was transferred from the white reference standard to the object of interest and a full spectrum scan was performed. Seawater reflectivity (albedo) was obtained from literature and satellite-based measures [27,28]. These data were applied to a heat balance equation: *Q*_in_ + *Q*_abs_ = *Q*_out_ + *Q*_st_, where heat in (*Q*_in_) is by long wavelength infrared thermal radiation (IR) from the sky and clouds, *Q*_abs_ is by solar radiation absorption, *Q*_out_ is by conduction, convection, evaporation and emitted IR, and *Q*_st_ is stored heat. We used these calculations to determine whether any observed differences between ambient (transmitter-derived) and satellite- or model-derived SST measures could be explained by reflectivity differences between ambient seawater and turtles, tags or *Sargassum*.

Finally, (iv) we measured and compared thermal profiles of seawater with and without *Sargassum* to verify that the *Sargassum* thermal environment is warmer than that of open seawater. Fresh *Sargassum* was collected offshore of southeast Florida placed in one of two identical buckets (28 cm inside diameter × 36.8 cm high) filled with filtered seawater. The buckets were each placed in circular plastic tubs (82 cm diameter × 15 cm high) filled with freshwater. Temperature was recorded every 30 min using Hobo U22 temperature data loggers (with ± 0.2°C accuracy; http://www.onsetcomp.com/products/data-loggers/u22-001). Data loggers were placed in air adjacent to the buckets, and each bucket had data loggers placed centrally, 2.5 cm below the water surface or water–*Sargassum* surface, and suspended at half-depth. We estimated percentage cloud cover visually; sunrise and sunset, wind speed and humidity were inferred from data at a nearby airport (less than 1 km away), and corrected for bucket height above the ground. Temperature data from the data loggers were overlaid to compare thermal profiles among treatments.

## 3. Results

### (a) Turtle movements

**Figure 1.**
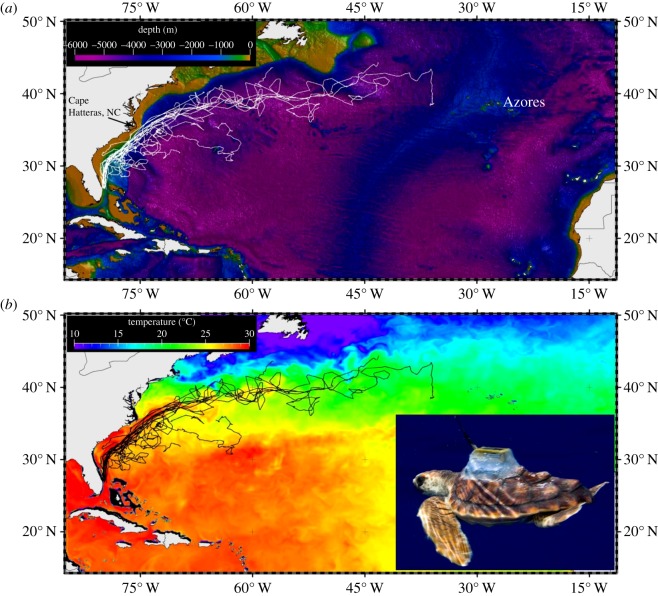
(*a,b*) Satellite tracks of neonate loggerhead sea turtles (109–281 days old) overlaid with bathymetric Gridded Global Relief Data, ETOPO2v2 (figure 1*a*; turtle tracks in white) and composite SST data (figure 1*b*; turtle tracks in black). Photo credit for figure 1 (*b*): J. Abernethy (2009).

Turtles were remotely tracked for between 27 and 220 days (mean = 86.6 days ± 55.2 s.d.; table 1) and travelled distances that ranged from 200 to more than 4300 km (figure 1*a*). All turtles initially travelled north, remaining within or in close proximity to the Gulf Stream immediately post-release. Ten turtles continued within the Gulf Stream, past Cape Hatteras (North Carolina, USA), then moved eastward into the northwestern Atlantic. Initially, turtles remained along the outer edge of Continental Shelf (defined by the 200 m isobath; figure 1*a*). Complete departure from near-shelf waters occurred past Cape Hatteras (approx. 35° N). One turtle spent approximately 21 days within Continental Shelf waters; however, 98.6% of all locations across all turtles (*n* = 1472 track days) were off the Shelf.

**Table 1.** Metadata for tracked turtles including turtle ID, straight carapace length (SCL) and total weight at time of release, sex, age of turtle, hatch date, release date, release location and track duration, and the number of days associated with each current feature: Gulf Stream, eddy feature and other. The Gulf Stream is defined as the main part of Gulf Stream not including eddy features. Eddy feature is defined as any current vector group forming a circular pattern. ‘Other’ includes the remaining locations (and days) not included in previous Gulf Stream or eddy groups.

turtle ID	SCL (mm)	weight (g)	sex	age (days)	hatch date	release date	release location	track duration (days)	Gulf Stream (days)	eddy (days)	other (days)	% eddy
92590a	150	577	F	247	04 Sep 2008	09 May 2009	26.883 N, 79.883 W	80	26	54	0	68
92584a	149.5	537	F	214	07 Oct 2008	09 May 2009	26.883 N, 79.883 W	39	19	20	0	51
92585a	182.8	615	F	251	04 Oct 2008	12 June 2009	26.829 N, 79.823 W	58	22	36	0	62
92587a	163	720.5	F	251	04 Oct 2008	12 June 2009	26.829 N, 79.823 W	56	15	41	0	73
92588a	169	692	F	281	04 Sep 2008	12 June 2009	26.829 N, 79.823 W	49	17	32	0	65
92586a	133.5	364	M	127	10 Aug 2009	15 Dec 2009	26.761 N, 79.823 W	39	17	22	0	56
92589a	146	475	F	127	10 Aug 2009	15 Dec 2009	26.761 N, 79.823 W	171	12	129	30	75
85512	117	314.8	F	109	30 June 2010	18 Oct 2010	26.292 N, 79.683 W	32	28	4	0	13
92585	121	309.5	F	109	30 June 2010	18 Oct 2010	26.292 N, 79.683 W	65	23	42	0	65
92586	129.9	315.4	F	114	11 July 2010	02 Nov 2010	26.761 N, 79.856 W	71	18	53	0	75
92587	132.8	338.3	F	127	28 June 2010	02 Nov 2010	26.761 N, 79.856 W	220	45	128	47	58
92590	124	329.6	F	117	08 July 2010	02 Nov 2010	26.761 N, 79.856 W	93	36	57	0	61
85511	114.8	297.3	F	141	14 July 2010	02 Dec 2010	26.738 N, 79.510 W	27	9	18	0	67
92588	124.9	339.5	F	168	20 July 2010	04 Jan 2011	26.750 N, 79.783 W	74	8	60	6	81
85513	119.5	328.3	F	173	14 July 2010	04 Jan 2011	26.750 N, 79.783 W	169	20	123	26	73
85514	122.6	337.2	F	155	02 Aug 2010	04 Jan 2011	26.750 N, 79.783 W	127	26	101	0	80
92584	133.3	390.6	F	168	20 July 2010	04 Jan 2011	26.750 N, 79.783 W	102	17	62	23	61

**Figure 2.**
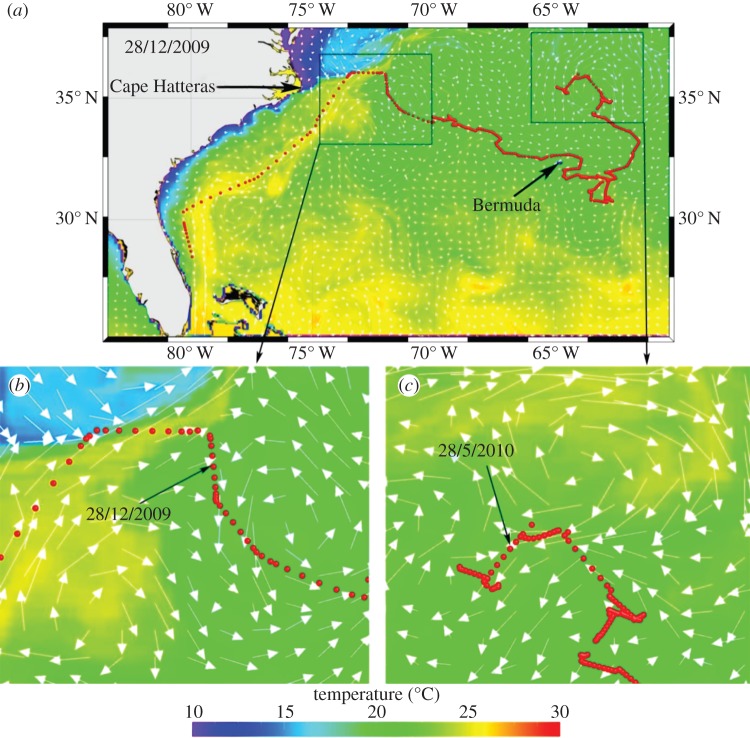
(*a*) Track of turtle (ID 92589_2009, red dots) overlaying temperature and current vectors from HYCOM model output on 28 December 2009. (*b*) A close-up view of track positions around 28 December showing two eddy features: one clockwise at lower left, the other counter-clockwise on the right. (*c*) A close-up view of track positions around 28 May 2010 showing the turtle between three eddy features: one anti-clockwise on the left, one on the bottom also anti-clockwise, and the other clockwise on the top.

All tracks showed significant directional movement (Raleigh’*s Z*; *p* < 0.05) throughout their tags’ transmission period. Turtles’ net paths were directed to the NNE–NE (38.9° ± 16.6 s.d.; *n* = 17) between release points to approximately 35°N latitude. Turtles travelled to the ENE (63.4° ± 21.4 s.d.; *n* = 11) within north Atlantic waters, north of 35° N latitude. With one exception, none of the turtles moved westward of the Gulf Stream boundary; however, turtles did move east beyond the eastern Gulf Stream boundary. Turtles spent 24.3% (± 9.3% s.d.) of their track time (*n* = 1472 days) within the main Gulf Stream current (table 1). Seven turtles travelled out of the Gulf Stream, moving into the Sargasso Sea (figure 1*b*). Some movements out of the Gulf Stream were associated with meso-scale eddies (table 1). Our current feature utilization analysis showed dominant utilization of eddy features (table 1 and figure 2*a–c*). Turtles spent between 13 and 81% of their time in association with eddies (mean: 66.7% ± 39.6 s.d.); typically along the edges of the meso-scale features (an example is shown in figure 2*a–c*).

Nine turtles reached 35°N latitude (*n* = 9) in 11–19 days from their release off southeast Florida while travelling in the Gulf Stream. Turtles continuing in the Gyre reached waters south of Georges Banks in 20–30 days, and waters off the Grand Banks (approx. 45°N, approx. 53°W) in 50–70 days. One turtle travelled to a point west of the Azores in 219 days prior to tag transmission cessation (figure 1*a*).

### (b) Thermal ecology and vertical habitat use

**Figure 3.**
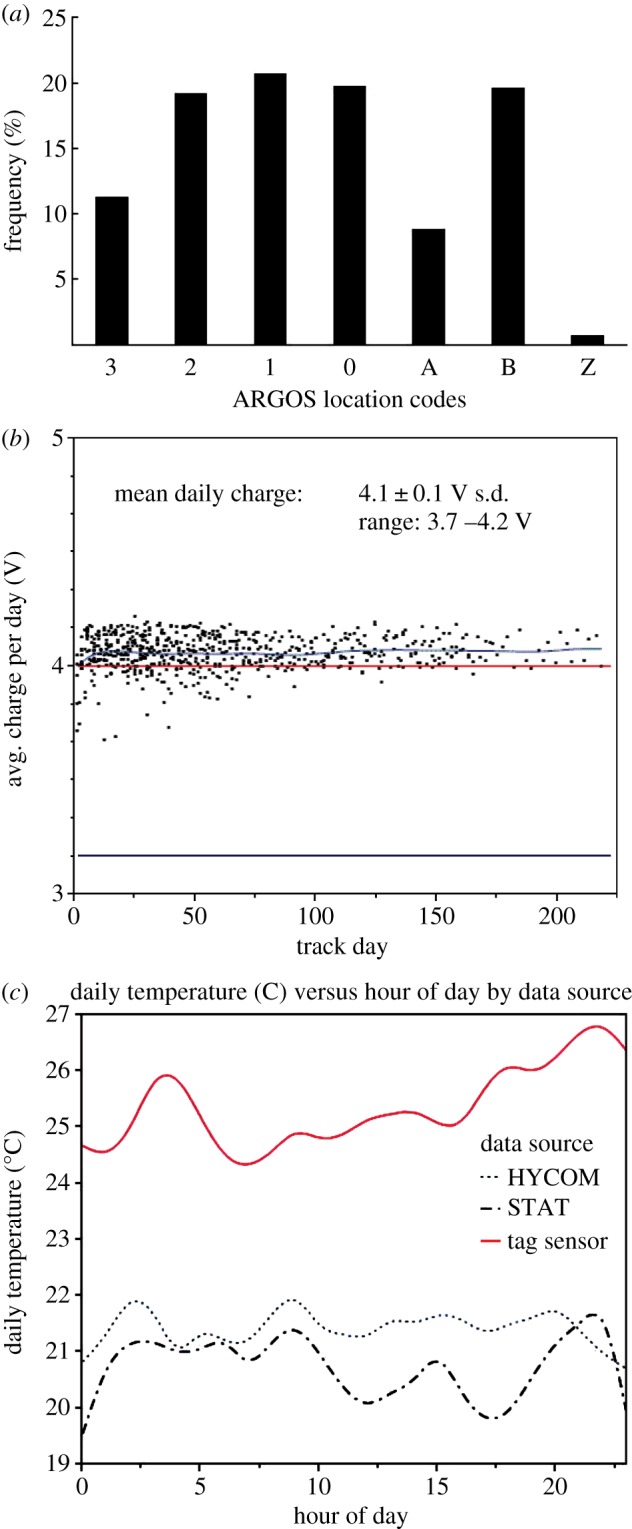
(*a*) Frequency (%) of Argos location codes reported with satellite track locations from neonate loggerhead sea turtles released in the western Atlantic. (*b*) Average daily solar cell charge (volt) reported from satellite tags (*n* = 17) deployed on neonate loggerhead sea turtles. Operational charge level (per manufacturer specifications) is represented by a black line (3.0 V) at the bottom of the graph; manufacturer-specified optimal charge (4.0 V) is represented by a red line, and mean daily observed charge (4.1 ± 0.1 V s.d.) is represented by a blue line. (*c*) Daily temperatures (°C) derived from satellite tag sensors (red line), HYCOM model (dotted line) and satellite-derived SSTs (STAT; dot-dashed line).

Argos location code accuracy, transmitter solar charging rates and temperature sensor data combine to allow for niche characterization. The majority of Argos location codes received were high quality: 77.2% of messages had high LCs of 0–3 (figure 3*a*). All tags maintained adequate operational charges (greater than 3.2 V) and optimal mean charges (greater than or equal to 4.0 V) throughout their transmission periods (figure 3*b*). These data combine to suggest that turtles were remaining at the sea surface. The tags’ high Argos location accuracy confirms that tag antennae were exposed to air and in communication with overhead satellites. Satellite-derived average daily SST encountered by the turtles was 20.8 ± 3.4°C and was similar to model-derived HYCOM average daily temperature of 21.4 ± 3.4°C (figure 3*c*). Internal tag temperature sensors recorded ambient temperatures that ranged from 17°C to 35°C (mean: 25.6 ± 3.7°C; figure 3*c*)—consistently averaging 4–6°C higher than temperatures derived from remote satellite data or HYCOM. This difference was statistically significant (Mann–Whitney *U*; *p* < 0.05). Optimal battery charging and the higher tag-recorded ambient temperatures compared with satellite- and HYCOM-derived SST data suggest that the tags’ solar cells were exposed to the sun's rays [7].

**Figure 4.**
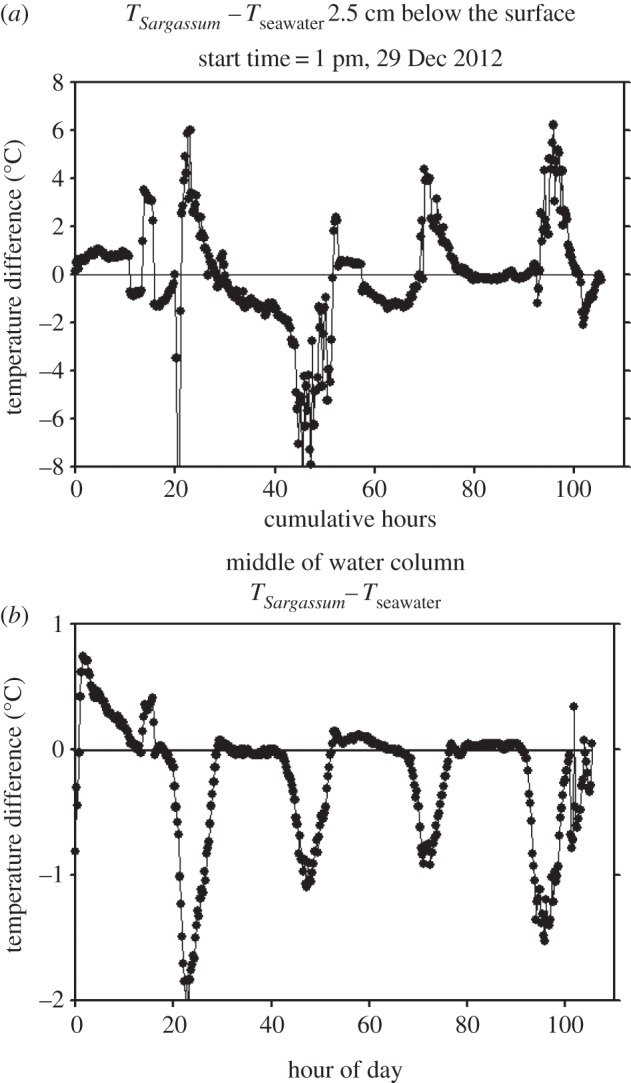
(*a,b*) Seawater temperature differences within paired containers of *Sargassum* mat versus plain seawater.

Reflectivity of the *Sargassum* (10–14%), tags (6–11%) and loggerhead turtle shells (7%) did not differ substantially. Empirical tests of seawater surface temperatures within buckets containing *Sargassum* mats versus plain seawater confirm the assumption that water temperature near the surface within *Sargassum* was consistently warmer when exposed to sunlight than in seawater without *Sargassum* (figure 4*a,b*). The *Sargassum* intercepted more solar radiation just below the surface where the water was consistently warmer than in the seawater bucket without *Sargassum* (figure 4*a*). The water column halfway to the bottom in the bucket without the *Sargassum* was warmer than the water temperature in the middle of the *Sargassum* bucket because more sunlight reaches deeper when there is no *Sargassum* to intercept it (e.g. figure 4*b*). As part of the *Sargassum* was above the water surface, night-time evaporation cooled it below the temperature of the seawater bucket during non-daylight hours.

## 4. Discussion

Our study provides the first successful satellite tracks for any neonate sea turtle. We also provide the first long-term empirical and *in situ* tracking data to characterize neonate loggerhead oceanic movements and surface habitat use. Tracked turtles rarely occupied Continental Shelf waters, supporting the loggerhead oceanic nursery paradigm. The turtles’ tracks were distinctly constrained east of the 200 m isobath, along the outer edge of the Continental Shelf. This off-shelf demarcation differs from the mostly on-shelf distributions and habitats used by larger, older, juvenile or sub-adult and adult loggerheads in western Atlantic waters [29].

Turtles travelled more variable routes than implied by the classic Gyre dispersal hypotheses [1,4]; rather, the turtles’ routes are more consistent with, and help validate, theoretical migratory trajectories derived by Putman *et al*. [30] using oceanographic models and experimentally derived navigation behaviour. While the Gulf Stream provided initial transport, turtles did not select the fastest or most direct routes to known oceanic developmental habitats (e.g. the Azores, Madeira or Cape Verde). Turtles instead travelled along net clockwise trajectories using a variety of paths. These paths indicate that dispersal is not uniformly unidirectional; deviations from outer Gyre currents and boundaries are common, invalidating previous hypotheses assuming a unidirectional developmental migration route following or entrained within the currents of the NASG.

The paths of our tracked neonate loggerheads were environmentally constrained. No turtles moved into lethally cold waters. Among the fraction of turtle tracks associated with the Gulf Stream or NASG current regime, net directional movements were consistent with the use of regional guideposts (e.g. magnetic cues) to orient the turtles along hypothesized routes [22]. Consistent with Lohmann & Lohmann [22], turtles did not travel beyond the constraints of the outer Gyre boundaries; orientation on a macro scale to remain within the Gyre boundaries is likely. However, deviations from the Gyre currents were oriented towards the interior of the Gyre. Turtles’ paths in and out of the Gulf Stream and Gyre currents as well as turtles’ association with meso-scale eddies imply that localized (‘micro-scale’) orientation and the duration of the turtles’ travels or regions they encounter can vary.

Some of our tracked turtles left the Gulf Stream and travelled into the Sargasso Sea, a behaviour that might be explained by the seasonal distribution of *Sargassum* in the northwestern Atlantic. *Sargassum* travels from the Gulf of Mexico along the eastern USA coast to the northwestern Atlantic before settling to the south as epipelagic mats in the Sargasso Sea [31]. Oceanic-stage turtles may opportunistically remain with those *Sargassum* habitats, leaving the Gyre currents and instead exploiting favourable foraging and thermal niches within the Sargasso Sea.

Neonate loggerheads can travel from southeast Florida to Azorean waters in less than a year, somewhat faster (e.g. approx. 220 days in the case of one turtle) than Carr's [4] drift bottle hypothesis suggests (235 days) despite the potential of slowed movement owing to tag effects (such as hydrodynamic drag). Mansfield *et al*. [7] demonstrate that biologically significant costs to the turtles from tag effects are minimal. This work compared neonate turtles with and without tags under controlled laboratory conditions over a period of several months. Mansfield *et al*. [7] found no significant differences in growth, condition, swimming behaviour and feeding among the test groups, suggesting that there are minimal energetic costs to the turtles due to the hydrodynamic effects of the tags [7]. Potential drag effects were mitigated by creating a teardrop attachment shape and placing the tags behind and between vertebral ‘spikes’. Using the techniques developed by Jones *et al*. [32], estimated drag may range as low as 4% or as high as 10% depending on turtle size (T. Jones 2014, personal communication). However, it is important to note that these estimates assume laminar flow conditions. The surface-based habitat occupied by the turtles is one in which the tags are likely to be out of the water, exposed to air, so that drag may be further minimized by two orders of magnitude [33,34]. There also remains the hypothesis that oceanic-stage sea turtles are passive drifters [1,4]. Working on the assumption that oceanic-stage turtles are, at a minimum, part-time passive drifters, then the net energetic cost due to drag would be further reduced. Finally, the in-water behaviour of the turtles tracked in this study was similar to that of larger, wild-caught turtles, suggesting some degree of natural behaviour. Specifically, the neonates tracked in this study showed a similar association with meso-scale eddies as larger, wild-caught subadult or neritic juveniles satellite tracked in the western Atlantic ocean [35].

By occupying oceanic surface layers, young turtles probably receive thermal benefits from solar absorption—either directly via their carapace at the air–sea interface, or indirectly through association with *Sargassum* or other flotsam. Tag sensor data, coupled with solar reflectivity tests, suggest that turtles are indeed occupying this air–sea interface, thus bolstering the thermal niche hypothesis. The tags’ high Argos location accuracy confirms that tag antennae were exposed to air and in regular communication with overhead satellites. Optimal tag battery charges indicate that the tags’ solar cells were exposed to the sun's rays [7]. The difference in the tags’ recorded ambient temperatures compared with satellite- and HYCOM-derived SST data (4–6°C) could be due to (i) biases owing to location, model and data resolution or error, and/or (ii) the thermal effects sun exposure. Surface seawater where neonate turtles are typically found [36] is highly transparent. Solar energy may be dissipated over a substantial vertical depth. However, *Sargassum*, neonate sea turtles and satellite transmitters have zero transparency and low reflectivity; thus, the absorption of solar radiation is concentrated near their respective surfaces. Furthermore, floating *Sargassum* mat structure impedes lateral water flow, thereby inhibiting convective transport of absorbed solar energy (heat) into the surrounding water. Under these conditions, energy retention can raise local water temperatures up to 6°C above that of surrounding water, as observed by ambient tag sensor data and as our bucket experiments demonstrated (e.g. figure 4*a*).

The thermal benefits that small sea turtles gain from remaining at the sea surface or associating with *Sargassum* communities probably differs somewhat from basking—a common thermoregulatory behaviour used by reptiles. Atmospheric basking (out of water) is common among reptiles, including turtles, for thermal regulation, as well as enhanced digestive efficiency, epibiont control and enhanced vitamin D synthesis [37,38]. A common feature of turtle basking, generally, is that it is episodic (not chronic as in the case of neonate turtles at the sea surface), being initiated when temperatures approach operative environmental temperatures [37,38]. Atmospheric basking is known in some populations of *Chelonia mydas* and results in increased body temperatures [39,40]. A single study of *Caretta caretta* found that loggerhead internal temperatures were higher when basking at the water's surface during sunny periods [41]. The authors attributed this increase in body temperature to increased absorption of solar radiation [42].

If exposed to sunlight, turtles’ shells will be likely to gain some degree of warmth. A surface-based, thermally driven developmental niche makes sense in a broader evolutionary context. Sea turtles exhibit a number of traits that natural selection probably acted on for a surface-based thermal developmental niche to have evolved. *Sargassum* habitats provide young, cold-blooded turtles with a thermal environment that promotes growth, eventually reducing the assemblage of predators capable of consuming them. Sea turtles are ectotherms; exposure to cooler habitats tends to reduce rates of food consumption and individual growth compared to exposure to warmer environments. Thermal differences and chronic or acute exposure to unfavourable temperatures can influence age and size at maturity in turtles [42,43]. Thermal habitat availability and early exposure to thermally beneficial developmental habitats probably has broad implications for age (or size) at neritic recruitment, particularly within different ocean basins or relative to different sea turtle rookeries. Exposure to UV radiation enhances reptilian calcium-dependent functions, including vitamin D-associated skeletal mineralization and growth [44]. Life at the surface also exposes turtles to airborne cues that can lead to the next patch of productive ocean [45]. Localized warming by only a few degrees can have significant impacts on temperature-dependent processes in reptiles, including digestion, growth and activity time. Availability of thermally beneficial habitat early in life can have important long-term impacts on the survival and fitness of sea turtles.

By combining persistent sea-surface-based behaviour with oceanic-stage turtles’ known association with *Sargassum,* we propose a new thermal niche hypothesis and possible mechanistic framework for why the sea surface and *Sargassum* habitats are important for the development and likely survival of oceanic-stage sea turtles.

## Notes

All research was conducted in compliance with the protected species laws of the United States and under Florida Atlantic University IACUC approval (A08–40), Florida Marine Turtle Permit (MTP-073) and US Fish and Wildlife Service Permits (USFWC-TE05217-2).

## Data accessibility

Data from this study are archived online using seaturtle.org's STAT (http://www.seaturtle.org/tracking/?project_id=717). These data are directly linked to the OBIS-SEAMAP data repository (http://seamap.env.duke.edu/provider/STAT).
